# Genome Aberrations in Canine Mammary Carcinomas and Their Detection in Cell-Free Plasma DNA

**DOI:** 10.1371/journal.pone.0075485

**Published:** 2013-09-30

**Authors:** Julia Beck, Silvia Hennecke, Kirsten Bornemann-Kolatzki, Howard B. Urnovitz, Stephan Neumann, Philipp Ströbel, Franz-Josef Kaup, Bertram Brenig, Ekkehard Schütz

**Affiliations:** 1 Chronix Biomedical, Göttingen, Germany; 2 Institute of Veterinary Medicine, Georg-August University Göttingen, Göttingen, Germany; 3 Department of Pathology, University Medicine, Göttingen, Germany; 4 Pathology Unit, German Primate Center, Göttingen, Germany; Utrecht University, The Netherlands

## Abstract

Mammary tumors are the most frequent cancers in female dogs exhibiting a variety of histopathological differences. There is lack of knowledge about the genomes of these common dog tumors. Five tumors of three different histological subtypes were evaluated. Massive parallel sequencing (MPS) was performed in comparison to the respective somatic genome of each animal. Copy number and structural aberrations were validated using droplet digital PCR (ddPCR). Using mate-pair sequencing chromosomal aneuploidies were found in two tumors, frequent smaller deletions were found in one, inter-chromosomal fusions in one other, whereas one tumor was almost normal. These aberrations affect several known cancer associated genes such as *cMYC,* and *KIT*. One common deletion of the proximal end of CFA27, harboring the tumor suppressor gene *PFDN5* was detected in four tumors. Using ddPCR, this deletion was validated and detected in 50% of tumors (N = 20). Breakpoint specific dPCRs were established for four tumors and tumor specific cell-free DNA (cfDNA) was detected in the plasma. In one animal tumor-specific cfDNA was found >1 year after surgery, attributable to a lung metastasis. Paired-end sequencing proved that copy-number imbalances of the tumor are reflected by the cfDNA. This report on chromosomal instability of canine mammary cancers reveals similarities to human breast cancers as well as special canine alterations. This animal model provides a framework for using MPS for screening for individual cancer biomarkers with cost effective confirmation and monitoring using ddPCR. The possibility exists that ddPCR can be expanded to screening for common cancer related variants.

## Introduction

The most common neoplasms in female dogs are mammary tumors representing more than 40% of all tumors diagnosed [1], [2]. An incidence rate of approximately 200/100,000 dogs/year is reported in studies conducted in the UK and Italy [3]–[5]. The risk of developing mammary cancer is significantly lowered by performing an ovari(ohyster)ectomy at an early age, resulting in lower incident rates in countries where this surgery is common practice [2]. In our own three-year clinical study an annual incidence rate of ∼1% in a cohort of 9,265 dogs, which were presented as patients in the Clinic for Small Animals in Goettingen, Germany, was calculated. Most mammary tumors in dogs are of epithelial origin, some consist of epithelial and myoepithelial tissues, termed complex carcinomas. Fewer tumors are of mesenchymal origin (e.g. osteosarcomas or fibrosarcomas), which frequently contain epithelial tissues (carcinosarcoma) [2].

Many similarities between cancers in humans and in dogs have been described, including the response to therapies, the incidences of different cancers, as well as environmental and personal risk factors [6], [7]. It is noteworthy that human and canine genomes have a higher similarity than the human and murine genomes [8]. Consequently, the dog has been emphasized as model animal for human cancers that is better suited than rodents for both studying the tumor biology and developing new drugs and therapies [7], [9].

Canine mammary tumors have been evaluated as model for human breast tumors, because in contrast to rodents, mammary gland tumors develop spontaneously in both dogs and humans. The shared risk factors are age, genetic predisposition as well as obesity in early life [2], and a hormonal etiology is described in both species [10]. In human breast cancers the expression of estrogen/progesterone receptors (ER/PR), the human epidermal growth factor receptor 2 (HER2/ERBB2) and basal or myoepithelial markers is routinely assessed. Malignant breast tumors are classified in the ER/PR positive types Luminal A (ERBB2 negative) and Luminal B (ERBB2 positive) and the ER/PR negative types: basal-like, ERBB2 positive, and normal-like. The same subtypes have been identified in canine mammary carcinomas, but are not routinely determined [11], [12]. The expression of ER/PR and the HER2 protein in human breast cancers is linked to prognosis and is relevant for therapeutic decisions [13]. ER and PR positivity is less frequent in canine primary cancers and metastases than in humans, indicating an earlier loss of hormone dependency in canine as compared to human tumors [14]. A decreased expression of ER was demonstrated in larger tumors and in lymph node involvement, associated with a worse prognosis. [15]. Tamoxifen a commonly administered anti-estrogen drug in humans has no anti-tumor effect in dogs [2], [16], and the response to certain chemotherapeutic drugs (e.g. Doxorubicin and Docetaxel) is different between human breast and canine mammary cancers [17]. Due to the unequivocal results on the prognostic value of ER/PR and HER2/ERBB2 in dogs, the tumor size, histological stage, invasive growth, lymph node involvement, and dedifferentiation are considered as the most important prognostic factors [2]. A direct comparison of the canine and human histological types of breast malignancies is difficult due to different classification schemes. However the human tubulopapillary carcinoma sub-type of invasive ductal carcinoma [13] is comparable to the simple tubulopapillary carcinoma in dogs, which is seen most frequently [18]. Strandberg and Goodman stated that canine infiltrating malignant epithelial neoplasms of duct origin are histologically very similar to those in women [19]. However, sarcomas and carcinosarcomas as seen in dogs to some extend are rare in humans [18]. Furthermore, canine mammary tumors often contain proliferating myoepithelial cells (mixed tumors) a condition also rarely seen in humans.

A hallmark of cancers is the occurrence of genomic structural aberrations including copy-number imbalances (CNIs) and copy-number neutral rearrangements [20]–[23]. In humans recurrent chromosome aberrations were reported that are shared by specific cancers [24]. In dogs, cytogenetic studies are complicated by the complex canine karyotype [25], therefore, the genome analysis of canine neoplasms greatly benefited from the development of CGH microarrays (aCGH) [26]. Also, copy-number differences in the germline of different breeds have been discovered by the use of aCGH [27]. In a recent aCGH study CNI regions with significant overlap between canine and human colorectal tumors have been described [23]. Chromosomal instability in human cancers is increasingly investigated by use of next-generation sequencing (NGS) [28]. The analysis of paired-end mapping (PEM) signatures obtained by NGS provides the potential to detect all aberrations including copy-number neutral rearrangements, together with their exact positions.

Cell-free nucleic acids (cfNA) in blood plasma were first described in 1948 by Mandel and Métais [29]. After the first detection of *KRAS* point mutations in the blood plasma of cancer patients in 1994 cfNA have been extensively studied especially with respect to the detection and monitoring of cancers [30]–[32]. Almost all manifestations of cancer-associated genomic, genetic and epigenetic variabilities have been investigated in cfDNA. These include microsatellite alterations, point mutations, alterations of repetitive elements, DNA methylation differences (for review see [30]). It has been shown that tumor-specific chromosomal breakpoints, segmental copy-number differences and quantitative differences of repetitive elements can be detected in the patients’ cfDNA [33]–[35]. Increasingly recognized is the fact that cell-free nucleic acids can provide a “liquid biopsy” that allows for genomic analysis of tumors from a simple blood draw. Recent studies have shown that by the use of high-throughput technologies such as SNP-microarrays and NGS a comprehensive picture of a tumor’s genome can be obtained from the cfDNA [36], [37].

In this article a broad overview of aberrations detected in five canine mammary carcinomas is provided. To our knowledge this is the first time that next-generation sequencing is applied to the genomic analysis of canine tumors. Furthermore, the cfDNA of the same animals was analyzed. It is shown that tumor-specific chromosomal breakpoints and CNIs can be detected in the cfDNA.

## Materials and Methods

### Clinical Samples

The five canine cases suffering from mammary carcinomas were patients in the Clinic for Small Animals of the Institute of Veterinary Medicine at the Georg-August-University of Goettingen. Tumors were surgically removed and the diagnosis was confirmed by histological evaluation and additional 15 samples were analyzed by ddPCR. Sample details including age at surgery, breed, tumor histology, and tumor stage are given in Table 1. Malignant tumors were selected to resemble the profile of cases as seen in the Clinic for Small Animals of the Institute of Veterinary Medicine. An ethical approval was not necessary according to the German Tierschutzgesetz (§7), because all samples were obtained as part of routine diagnostic procedures and with informed owner consent; tumor samples were obtained by a veterinarian during the medically necessary surgeries.

**Table 1:** Information about breed, spaying status and age of the patients.

Tumor	Breed	Spayed	Age	Tumor Class	TNM
30	Jack Russell Terrier		13	Osteosarcoma	T3NXM0
35	Border Collie		12	Simple tubulopapillary	T3NXM0
40	Golden Retriever	+	10	Simple tubulopapillary	T1N0M0
47	French Bulldog		6	Simple tubulopapillary	T1NXMX
49	Berger Bl. Suisse	+	9	Complex carcinoma	T2NXMX
52	German Shepherd		9	Simple tubulopapillary	T1NXMX
60	Mongrel		11	Complex carcinoma	T1N0M0
65	German Spaniel		9	Complex carcinoma	T1N0M0
69	Mongrel		14	Simple solid	T3NXM0
73	Golden Retriever		5	Simple solid	T3N0MX
78	Mongrel	+	11	Simple tubulopapillary	T1N0M0
81	German Hound		13	Simple solid	T1N0M0
83	Mongrel		15	Simple tubulopapillary	T3N0M1
86	German Sh-h. Pointing		7	Simple solid	T1N0M0
92	Golden Retriever		10	Carcinosarcoma	T3N0M0
97	Labrador Retriever		7	Simple tubulopapillary	T3N0M0
98	Mongrel		7	Simple tubulopapillary	T3N0M0
99	German Shepherd		5	Complex carcinoma	T3N0M0
104	Mnt. Scenthound		15	Simple tubulopapillary	T3N0MX
108	Pudelpointer		8	Simple carcinoma	T3N0MX

Histological classification and stage of the tumors.

### Immunochemistry

For immunochemical assessment of the ER expression and the Ki-67 proliferation index the antibodies: ER alpha (EP1) (pH6, 1∶100, Epitomics) and Ki-67 (Ki67) (pH6, 1∶200, Zytomed Systems) were used. For evaluation of ER expression, the scoring system according to Allred D et al. was used [38]. The Ki-67 proliferation index was determined by counting 300 proliferating and non-proliferating cells in the area with highest nuclear labeling and the percentage of proliferating cells was calculated.

### Specimen Sampling

5–6 mL of blood was drawn from each animal. The blood was immediately centrifuged at 4000×g for 15 min at 4°C. The plasma supernatant was transferred into fresh tubes and stored at −20°C until extraction of the cfDNA. The peripheral blood mononuclear cells (PBMC) were also sampled and stored at −20°C until extraction of the genomic DNA. After surgical removal of the tumor, several radial cut pieces of the tumor tissue were homogenized.

### Extraction of Cell-free Plasma Nucleic Acids

In order to remove any cellular debris 0.7 mL plasma was re-centrifuged at 4000×g for 20 min and the supernatant was carefully removed. The cfDNA was extracted from 0.6 mL of plasma using the High Pure Viral Nucleic Acid Extraction Kit (Roche Applied Sciences) according to the manufacturer’s instruction, without the use of carrier RNA.

### Genomic DNA Extraction from Tumor Tissue and Blood Cells

Genomic DNA was extracted from 25 mg of homogenized tumor tissue using the QIAGEN Blood and Tissue Kit (QIAGEN) following the manufacturer’s instructions. Genomic DNA from the PBMC was extracted by a modified procedure according to Miller [39].

### Library Preparation and Sequencing

Mate-pair libraries were generated for the SOLiD4+ system (Lifetechnologies) as described elsewhere [40]. In brief, genomic DNA was sheared into ∼3 kb fragments and after circularization, nick-translation and adapter ligation used as template in emulsion PCR. The ligated adapters contained molecular barcodes. Sequencing of 40 bp for read 1 and 50 bp for read 2 was carried out on one SOLiD4+ slide. In addition, paired-end libraries were generated from two of the tumors, two corresponding cfDNA samples and five PBMC control genomes (50 bp read 1 and 35 bp read 2) (Table 2).

**Table 2:** Specimen overview with sequencing type, number of reads, mean insert size, haploid and insert coverage.

Specimen	Sequencing Type	Number Reads	Mean Insert Size (SD)	Haploid Base Coverage	Insert Coverage
Tumor 49	Mate-pair	52958301	2554 (719)	0.9	22
PBMCs 49	Mate-pair	46573332	2331 (638)	0.8	18
Tumor 47	Mate-pair	37059440	2415 (744)	0.6	15
PBMC 47	Mate-pair	42991422	2381 (620)	0.7	16
Tumor 30	Mate-pair	28996426	2013 (748)	0.5	10
PBMC 30	Mate-pair	35798374	2349 (612)	0.6	14
Tumor 52	Mate-pair	28435620	2593 (735)	0.5	12
PBMC 52	Mate-pair	22915754	2279 (679)	0.4	9
Tumor 35	Mate-pair	29418741	2269 (780)	0.5	11
PBMC 35	Mate-pair	47255421	2487 (655)	0.8	19
Tumor 49	Paired-end	88240507	88 (55)	1.2	1.4
PBMC 49	Paired-end	64811016	90 (49)	0.9	1.0
cfDNA 49	Paired-end	30960138	145 (67)	0.4	0.8
Tumor 47	Paired-end	86628229	128 (66)	1.2	2.0
PBMC 47	Paired-end	98267227	136 (85)	1.3	3.2
cfDNA 47	Paired-end	14857474	129 (21)	0.2	0.5
PBMC40	Paired-end	98964511	144 (99)	1.3	3.2
PBMC52	Paired-end	65341166	89 (42)	0.9	2.1
PBMC27	Paired-end	86829099	133 (65)	1.2	2.8

Sequence data were mapped to the canine genome reference sequence (canFam 2, May 2005) using the Bioscope (Lifetechnologies) software pipeline. Duplicate and low quality reads were removed using the PICARD tools (http://picard.sourceforge.net).

### Comparative Depth of Coverage (DOC) Analysis

The program CNV-seq was used to calculate ratios (expressed as log2) and p-values for each tumor/PBMC pair using globally normalized read densities in sliding windows [41]. The confidence level used to calculate the minimum window size for each sample was set to p = 0.0001 (p’) and a ratio of log2 = 0.6 (r’) and the final windows size was set to 4 times the minimum size. The obtained ratios were subjected to Parzen-Rosenblatt smoothing [42], [43]. The significance of any given value was corrected for each chromosome by the method for controlling the false discovery rate as suggested by Benjamini and Hochberg based on a 0.0001 confidence limit [44]. The resulting significance limits of the ratios (log2) for the different tumors were T49: −0.32>log2>0.32, T47: −0.29>log2>0.29, T30: −0.29>log2>0.29, T52: −0.31>log2>0.31, T35: −0.35>log2>0.35. These values were used to color significant copy-number imbalances in the Circos plots, which were built using the Circos software [45], and are basis of the indicators in Table 3. The genes given in Table 3 were selected from the significant focally amplified/deleted regions from published human breast cancer [24], [46] and was complemented by additional known cancer genes [47], [48].

**Table 3:** Genes frequently affected by copy-number changes in human breast cancer together with their location in the canine genome (canFam 2.0) and their copy-number status in four sequenced tumor genomes.

Gene	Cytobandhuman	Position dog	T30	T47	T49	T52
**Gain**						
MCL1	1q21.3	17∶62839120–62842626	↓			
PIK3CA	3q26.32	34∶15608269–15684188	↑			↑
EGFR	7p11.2	18∶8980893–9029252	↑	↓		↓
ZNF703	8p11.23	16∶30624187–30626955		↑		↓
MYC	8q24.21	13∶28240103–28242545	↑			
CCND1	11q13.3	18∶51527946–51535735	↑	↓		↓
MDM2	12q.15	10∶13920606–13946580				
FOXA1	14q21.1	8∶19085869–19089654				
IGF1R	15q26.3	3∶44883005–44947123	↓	↓		
HER2/ERBB2	17q12	9∶26088707–26112823	↑			
CCNE1	19q12	1∶124626035–124634636	↑	↑		
ZNF217	20q13.2	24∶42397068–42405906	↑			
NF1	17q11.2	9∶44796408–45004875	↑	↑	↓	↑
TERT	5p15.33	34∶14294248–14312666	↑	↑	↑	↓
FGFR1	8p11.22-23	16∶29991434–30031766	↑			↓
PPM1D	17q23.2	9∶39177916–39235090		↑		
PTK2	8q24.3	13∶38344934–38584509	↑	↑		
**Loss**						
FOXO3	6q21	12∶68584815–68688059	↓			
MLL3	7q36.1	16∶18911504–19079411				
CSMD1	8p23.2	16∶58080357–58442179				↓
PTPRD	9p24.1-23	11∶32127787–32436346		↓		
CDKN2A/B	9p21.3	11∶44291167–44294900		↓	↓	
PTEN	10q23.31	26∶40903727–40981745	↓			↑
RB1	13q14.2	22∶6005221–6146221	↓		↓	
WWOX	16q23.1-23.2	5∶75970696–76249161		↓		
MAP2K4	17p12	5∶39136226–39233459	↑	↓		↓
STK11	19p13.3	20∶60701050–60719499	↑	↑	↑	↓
BRCA2	13q13.1	25∶10719191–10782555				
TP53	17p13.1	5∶35557006–35560762	↑	↓		↓
BRCA1	17q21.31	9∶23278875–23342346	↑			
NF2	22q12.2	26∶25847427–25923360	↓	↑		

T35 was omitted because no conclusive aberrations were detected.

### Paired-End Mapping (PEM) Signature Analysis

Mate-pair mapping data were analyzed for aberrant PEM signatures using the program SVDetect [49]. A PEM signature is considered as anomalous if the pairs are not mapped in the expected distance, order and/or orientation. The threshold for deletions and duplications was set to 4 standard deviations (SD) from the mean library insert size as estimated by mapping the read pairs to the canine reference genome. The minimum number of supporting reads was set to 3 for the tumor samples and set to 1 for the PBMC samples. Strand and order filtering was conducted during final cluster calling. Putative aberrations detected in the tumor data were filtered out if equivalent structural variations were detected in the corresponding PBMC sample. The minimal overlap required for filtering was set to 1 bp. Additional filters were applied in order to remove false positive calls. For that purpose a perl script was written that searches for all clusters within a window of ±3 kbp in the control genomes (WindowFilter). If both links of a predicted tumor aberration were found within the given window of a PBMC structural variation, the cluster was filtered out as false positive. A link is defined as the cluster of reads defining one side of a aberration. Each aberration consists of two supporting links each containing the single reads of a minimum of 3 concordant read-pairs. All aberrations predicted for each tumor were filtered against the PBMC data of the respective animal (WindowFilter1) and subsequently against data obtained from the total set of PBMC genomes analyzed (WindowFilter2). The WindowFilter perl script is provided as Skript S1.

### Digital PCR Assays

#### Detection of the CFA27 proximal deletion

For the assessment of a deletion of the proximal end of chromosome CFA27 in the tumor genomes, a ddPCR assay was designed. 1 µg of genomic DNA of the tumors and corresponding PBMCs was digested with 5 units of *Apo*I restriction endonuclease in a total volume of 25 µL. ddPCR was performed using a QX100 Droplet Digital PCR System (Bio-Rad). Reaction mixture (20 µL) contained 2× ddPCR Master Mix (Bio-Rad), 900 nmol/L of each PCR primer, 250 nmol/L of hydrolysis probes and 60 ng DNA. Primers were designed ∼1,700 bp apart from the annotated *PFDN5* gene (amplified fragment: 4,915,227–4,915,298, PFDN5 location: 4,910,008–4,913,488 on canFam2 reference genome). The sequences of the primers and probe were: CFA27.F CAGGTGCAGCCCCAATAAGA, CFA27.R CCCCGCTTCTGTACTACGTC and CFA27-Probe 6-FAM-TTGAGTCGGGGAGCCTGGCG-BHQ1. A fragment amplified from chromosome CFA32 served as control amplicon. As judged from the DOC analysis the region had equal copy-numbers in each PBMC and tumor genome pair. The CFA32 primer and probe sequences were: CFA32.F AAAAGCCTCCAATCCCCGAG, CFA32.R CCTGACAGAAAAAGCAGCCC and CFA32-Probe HEX-CTCCGTGACAAGTCAAGCTCAATAGCCT-BHQ1. Each 20 µL PCR reaction was dispersed in a water-in-oil emulsion in order to generate ca. 20,000 droplets using QX100 Droplet Generator (Bio-Rad). Thermal cycling conditions were 95°C for 10 min followed by 50 cycles of 95°C for 30 sec and 62°C for 1 min. After thermal cycling the PCR plates were transferred to the QX100 Droplet Reader (Bio-Rad). The average number of droplets read for each ddPCR was 14,121 (SD: 995). The copy-number of the respective template fragments and the relative standard uncertainty was calculated based on the Poisson distribution using the Quantasoft™ software [50]. The estimated copy-number of the tested CFA27 fragment close to the *PFDN5* gene locus was calculated in relation to the reference fragment.

#### Detection of HER2/ERBB2 amplification

HER2/ERBB2 amplification was detected using the primers: ERBB2.F GAGACAGCCCCTTCACAC, ERBB2.R TGGATGTTCGCACTGGTG and the hydrolysis probe ERBB2-Probe 6-FAM-CTACGGTCTGGGCATGGAGCAC-BHQ1 with CFA32 amplicon as reference. The ddPCR was performed as described above using 50 ng of sheared DNA for each reaction and T_ann/ex_ was 58°C, 75 sec. For each animal the copy-number difference between the tumor and the PBMC DNA was calculated. Tumor DNAs with no amplification detected by sequencing had a ddPCR maximal copy-number difference of 0.02, all tumors with differences above this value were judged to harbor the amplification.

#### Quantification of individual rearrangements

Further, digital PCR assays were developed for the detection of tumor specific rearrangements in the tumor genomic DNA and the cfDNA. The sequence and position of the primer pairs, each spanning one particular rearrangement of one of the five tumors, are given in Table S1. A fragment of the *FGF5* gene located on CFA32 served as control amplicon. Primer sequences were: FGF5.F GAGAGGTAGTGAGAAGGTCAAAG, FGF5.R ACAATTCACATTATGGATGCCAAG. The PCR reactions were performed in a total of 5 µL including 0.25 units of Faststart DNA Polymerase (Roche), 200 µmol/L dNTPs, 500 nmol/L of each primer. DNA template was added to the reactions in a concentration equal to 0.5 haploid genome equivalents per well (1.3 ng total). Thermal cycling conditions were 95°C for 10 min followed by 50 cycles of 95°C for 30 sec, 62°C for 30 sec and 72°C for 30 sec. PCR was carried out on a Light Cycler 480 (Roche) instrument in a 384-well plate using EvaGreen for amplification detection. One half of a plate contained the respective rearrangement specific primers and the other half contained the *FGF5* reference primers. The DNA template was added to the mastermix, which was subsequently split before the two different primer pairs were added. Initially, the specificity of the amplification was verified by agarose gel analysis. The copy-number of each template molecule (breakpoint and reference) was calculated based on the Poisson distribution. The ratio between the respective breakpoint template and the *FGF5* template was calculated for the tumor, PBMC and cfDNA.

### Z-Score Calculation from Paired End Sequencing Data

Paired-end sequencing was conducted for 2 tumors (49 and 47), the 2 corresponding cfDNA and PBMC specimens and additional 3 PBMC samples (control group) (Table 2). The number of unique reads in windows of 5 Mbp was obtained and normalized to 10 M reads per sample. The Z-values for each tumor and cfDNA sample were calculated based on the mean and SD of the 5 PBMC samples. For chromosomal regions, which were identified as deleted or amplified by the mate pair data analysis (CNV-seq, Circos-Plot), the tumor Z-scores were compared to the cfDNA Z-scores by calculating the Pearson correlation coefficient and the respective F-statistic.

### Comparison to Human Published Data

For comparison of the prevalence of structural rearrangements between human and canine mammary cancers the human data were compiled from the supplementary material of publications by Stephens et al., Ellis et al., Nik-Zainal et al. and Shah et al. [51]–[54].

## Results

### Sequence Coverage and Mutation Analysis

Mate-Pair sequencing of DNA from five primary tumors and corresponding PBMC samples yielded 37 million mappable reads on average. The lowest number of reads was obtained for the tumor and PBMC of animal 52 (28.4 M and 22.9 M) the highest number of reads were obtained for the T49 (52.9 M) and additional paired-end sequencing generated 88 M, 87 M, 65 M and 98 M reads for T49, T47, PBMC49 and PBMC47, respectively. For the cfDNA of animal 49 and animal 47 a total of 31 M and 15 M reads were obtained. Table 2 summarizes the sequencing method, the number of reads, insert sizes, haploid base coverage and insert coverage for each of the specimens. Binary Alignment Map/BAM files with duplicates removed were submitted to the European Nucleotide Archive (study accession: PRJEB3952).

### Depth of Coverage Analysis

**Figure 1:**
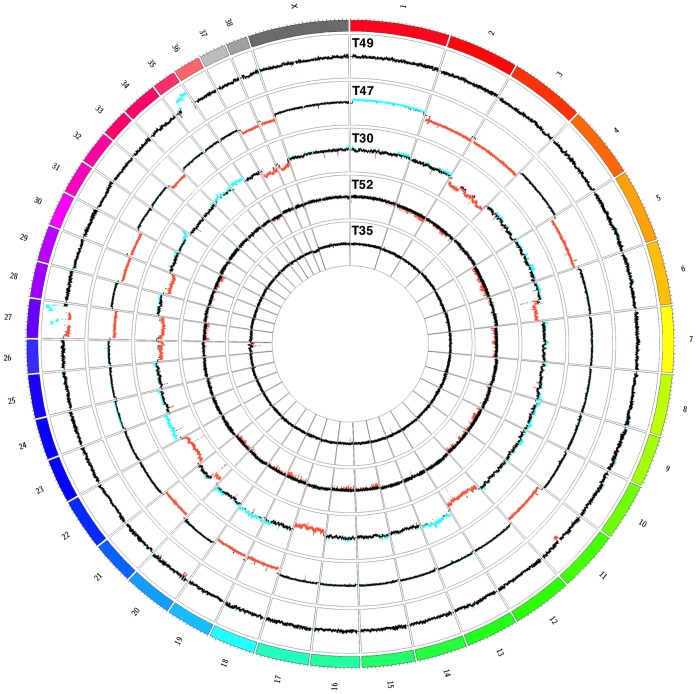
Circos Plot of detected copy number changes in the five sequenced tumor genomes. The outer track represents the canine ideogram, with each tick indicating 5-number profile of the tumors. Displayed are the ratios (as log2) of each tumor/PBMC pair. Regions with significantly negative log2-ratios (deletions) are highlighted in red and regions with significantly positive log2-ratios (amplifications) are highlighted in blue. Each data track’s y-axis spans from −3 to 3.

Copy number profiles of the five tumor samples were generated using the program CNV-seq and expressed as copy number ratio; window sizes ranged between 37,969 for animal 49 and 75,380 for animal 52 (Figure 1) Several high amplitude changes of the tumor/PBMC ratios were detected for two chromosomes CFA27 and CFA35 in T49. Both chromosomes contained intermittent and complex patterns of high-level amplifications and deletions. Furthermore, a deletion of a region between 42.6 Mbp and 47.9 Mbp on CFA11 affecting the tumor suppressor gene cluster *CDKN2A-CDKN2B* was detected. The same gene cluster was deleted in T47.

It was apparent that chromosomes of T49 were “shattered”, whereas the genome of T47 was characterized by hypoploidy with complete losses of 13 different chromosomes (CFA2, CFA3, CFA5, CFA11, CFA18, CFA19, CFA21, CFA27, CFA29, CFA30, CFA33, CFA37, CFA38) and one gain of the entire chromosome CFA1.

The highest number of copy-number changes was detected in the genome of tumor T30. It shared some similarities to T47 as both tumors showed losses of the chromosomes CFA27, CFA29, CFA37 and CFA38. Several large deletions were present on chromosome CFA3 of T30, while in T47 the loss affects the entire chromosome. Chromosome CFA13, which harbors the oncogenes *cMYC* and *KIT*, was amplified. Among the five sequenced tumor genomes only T30 was found to harbor at least two copies of the *HER2/ERBB2* gene (chr9∶26.08 Mb–26.11 Mbp).

The copy number changes in the sample T52 were less pronounced and not clearly confined. Only four small deletions, which most probably occurred due to mapping artifacts, were detected in the genome of T35.

The three tumors of simple tubulopapillary type (T47, T52, and T35) did not share substantial commonalities, except a slightly higher abundance of deletions over amplifications in the genomes of T47 and T52. Full lists of all regions with identified copy-number aberrations and the canine RefSeq genes located in these regions are provided as Tables S2–S5.

Table 3 lists published genes frequently affected by CNI in human breast cancer [24], [46]–[48] together with the copy-number status obtained in the sequenced canine tumor genomes. Copy-number changes concordant to human breast cancer can be recognized, e.g. gains in at least three tumors were detected for *TERT* and *NF1*. Furthermore, in accordance with human data *TP53*, *MAP2K4* and *CDKN2A/B* were deleted in two of canine tumors. On the other hand, some genes that are described as recurrently amplified in human breast cancers were affected by losses in at least two tumors (e.g. *EGFR*, *IGFR*, and *CCND1*). *STK11* is frequently lost in human breast cancers but was found amplified in three of the five canine tumors.

### Structural Aberrations Detected by Aberrant Read Pair Signatures

In addition, structural aberrations were predicted from PEM signatures using the program SVDetect [49]. By careful inspection of the resulting putative aberrations, we found many tumor read clusters with PBMC equivalents located in close proximity. To reduce the number of such false positive clusters we developed and applied an extended filter: The aberrations predicted for each tumor were filtered against the control genome of the respective animal (WindowFilter1) and subsequently against all control genomes (WindowFilter2). The numbers of predicted aberrations after each filter step are given in Table 4, which shows the improvement of the specificity of the aberration prediction.

**Table 4:** Number of predicted aberrations after SVDetect and additional filter steps in the five different tumor samples.

Animal ID	SVDetect	WindowFilter1	WindowFilter2
49	553	200 (36.2%)	35 (6.3%)
47	237	115 (48.5%)	5 (2.1%)
30	183	96 (52.5%)	34 (18.5%)
52	625	445 (71.2%)	7 (1.1%)
35	89	27 (30.3%)	1 (1.1%)

For the additional two filter steps the percentages of the aberrations initially predicted by SVDetect are given in brackets.

PCR primers were designed for i) 27 aberrations that remained after WindowFilter1 but were removed by WindowFilter2 and ii) all 82 aberrations that remained after WindowFilter2. Every primer pair was tested on the tumor and PBMC DNA of the respective animal and scored as validated if only tumor showed a PCR product. The validation rate for the total set of 109 aberrations was only 50% after WindowFilter1, but raised to 66% for those remaining after WindowFilter2. Especially the number of falsely detected germline structural variants was lowered from 39% to 24% by application of WindowFilter2. None of the validated aberrations was falsely removed by this more stringent filtering. All validated chromosomal breakpoints and the corresponding primer pairs are given in Table S6.

The numbers of validated aberrations for all five tumors are given in Table 5. In total we identified 54 somatic rearrangements. Consistent with the copy-number analysis the genomes of tumors T49 and T30 contained the most aberrations. Only one translocation was detected for T47. In the genome of tumor T52 we detected one insertion and one translocation. No chromosomal breakpoints were found in the genome of tumor T35. The prevalence of structural rearrangements in comparison to published data from different large scale sequencing studies of human breast cancers is given in Table 6 [51]–[54]. In the canine cancers less structural rearrangements were detected. The quantity of structural rearrangements in different tumors is heterogeneous in dogs and humans and tumors without such aberrations are found in both species.

**Table 5:** Overview of the validated chromosomal rearrangements detected by PEM signature analysis.

TUMOR ID	T49	T47	T30	T52	T35
TOTAL	23	1	28	2	0
LARGE DUPLICATION	2	0	3	0	0
INSERTION	0	0	0	1	0
DELETION	5	0	20	0	0
TRANSLOCATION	9	1	0	1	0
INVERSION	7	0	5	0	0

**Table 6:** Comparison of the prevalence of structural rearrangements detected in canine tumors and published data on human primary breast tumors.

	Canine Tumors	Human Primary Tumors			
		Stephens et al.	Ellis et al.	Nik-Zainal et al.	Shah et al.
**Number Tumors**	5	15	46	21	15
**Duplikation/Amplification**	1 (0–3)	45 (0–199)	na	21 (0–99)	na
**Insertion**	0.2 (0–1)	na	na	na	na
**Deletion**	5 (0–20)	10 (0–41)	13 (0–176)	12 (0–48)	na
**Translocation**	2 (0–9)	6 (0–27)	4 (0–29)	14 (0–74)	na
**Inversion**	2 (0–7)	7 (0–18)	0.1 (0–2)	9 (0–21)	na
**Total**	11 (0–28)	68 (1–231)	17 (0–178)	57 (2–217)	na
**Genefusion**	0.2 (0–1)	2 (0–7)	na	na	1.7 (0–6)

The occurrence per tumor is given as mean over all samples for the respective study (values >1 are rounded to integers, values <1 are rounded to one digit). Numbers given in brackets denominate the minimum and maximum values. Data for human primary tumors was derived from the supplements of references [51]–[54].

**Figure 2:**
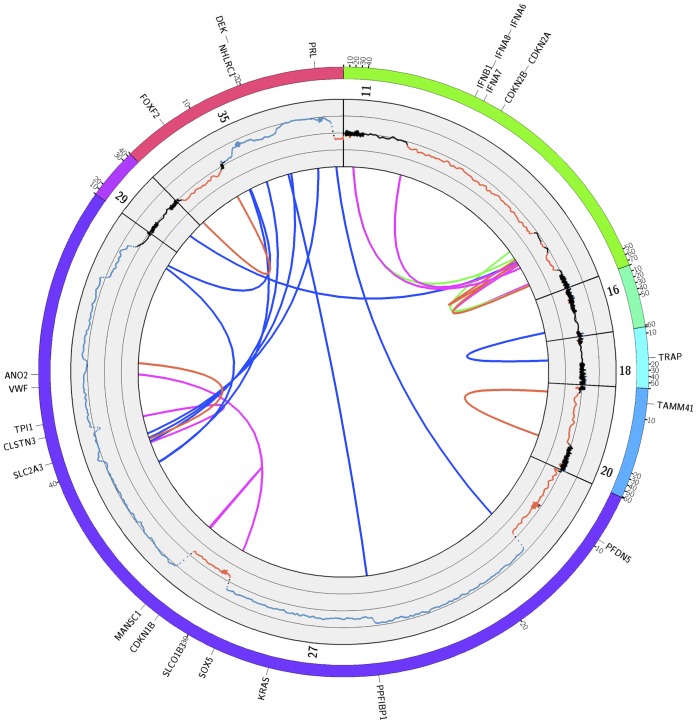
Structural aberrations detected in the genome of tumor T49. The outer track represents chromosomes with detected aberrations, with each tick indicating 10-number profile and the rearrangements identified by PEM analysis. The inner track displays log2 ratios as obtained by DOC analysis. The y-axis spans from −4 to 4 with sub-scales at −2 and 2. Arcs indicate the rearrangements detected by PEM analysis. Blue = translocations, red = deletions, green = duplications, magenta = inversions. For better visibility regions with rearrangements are expanded. Canine RefSeq genes affected by aberrations and/or copy-number imbalances are indicated.

Figure 2 shows the Circos plot with the combined copy-number and read pair data for tumor T49. Consistent with the DOC results a pronounced clustering of breakpoints is detected on chromosomes CFA27 and CFA35. Several interchromosomal translocations occurred between these two chromosomes. The affected regions showed patterns of complex rearrangements together with frequent changes in copy-number states. Several genes within the complex rearrangements on chromosome CFA27 were disrupted by translocations (*PPFIBP1*, *SLC2A3*) or inversions (*SOX5*, *ANO2*, *MANSC1*, *CLSTN3*). While *SOX5* and *ANO2* were fused by an inversion, no annotated fusion partners were detected for the other aforementioned disrupted genes. The SOX5:ANO2 fusion was located in introns 4 and 11 and was unlikely to produce a fusion protein, because of the inverted ORF of *ANO2*. Another region with a complex rearrangement pattern is located on chromosome CFA11∶42.62–47.92 Mbp around the tumor suppressor gene cluster *CDKN2A-CDKN2B*.

**Figure 3:**
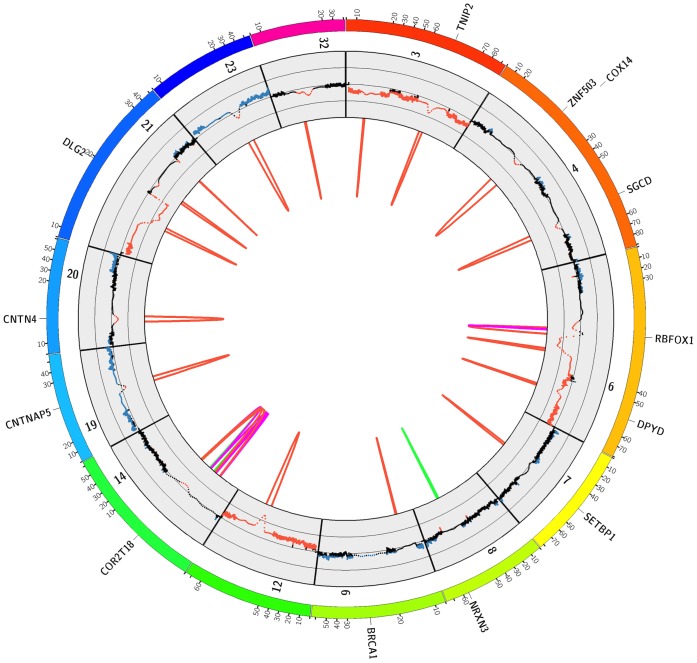
Structural aberrations detected in the genome of tumor T30. The outer track represents chromosomes with detected aberrations, with each tick indicating 10-number profile and the rearrangements identified by PEM analysis. The inner track displays log2 ratios as obtained by DOC analysis. The y-axis spans from −2 to 2 with sub-scales at −1 and 1. Arcs indicate the rearrangements detected by PEM analysis. Blue = translocations, red = deletions, green = duplications, magenta = inversions. For better visibility regions with rearrangements are expanded. Canine RefSeq genes affected by aberrations and/or copy-number imbalances are indicated.

A prevalence of smaller sized focal deletions characterized the genome of tumor T30 (Figure 3). One complex rearrangement was detected on chromosome CFA14. Many of these deletions resided in regions with haploid chromosome states, thus resulting in homozygous losses of the respective locus. The genes affected by the focal deletions are: *TNIP2*, *ZNF503*, *SGCD*, *RBFOX1*, *DPYD*, *SETBP1*, *CNTNAP5*, *CNTN4* and *DLG2*.

### Validation and Genotyping of a Recurrent Deletion of a Region on CFA27

The five tumor genomes are heterogeneous, with most of the detected aberrations not shared by more than two tumors. Only one recurrently deleted region at the proximal end of chromosome CFA27 was found in four out of five tumor genomes.

The commonly deleted region (ca. 8.8 Mbp) was confined by the smallest deletion that was detected in tumor T52. Several canine reference genes are annotated in this region. These comprise the olfactory receptor genes *COR6C13*, *COR6C47 COR9K3*, the keratin genes *KRT76*, *KRT1*, *KRT2*, *KRT71*, the nuclear receptor subfamily member 4 (*NR4A1*), the chymotrypsin-like elastase family member 1 (*CELA1*) and the adenylate cyclase type 6 (*ADCY6*) genes. Only two cancer associated genes *PFDN5* and *TMBIM6* were found in the deleted region. The BAX inhibitor 1 (*TMBIM6*) acts as oncogene by inhibiting apoptotic cell death and its expression is upregulated in human breast cancer [55]. Only the prefoldin subunit 5 gene (*PFDN5*) has previously been described as a tumor suppressor gene [56] and, therefore, is the most conclusive candidate for possible functional impact.

**Figure 4:**
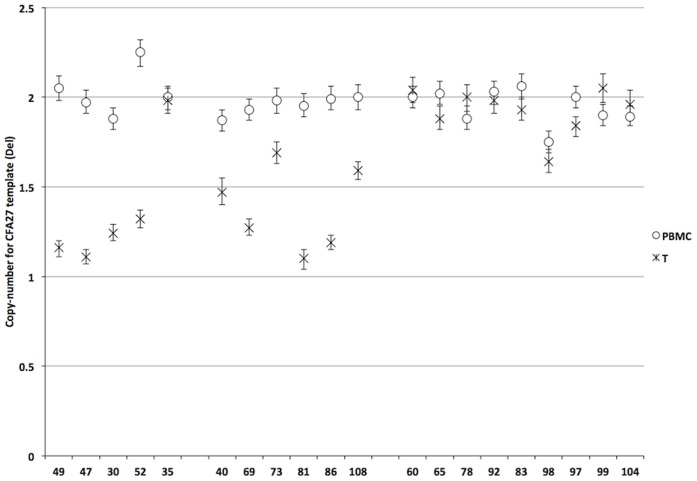
CFA27 proximal region copy-number states as detected by ddPCR. Left: Copy-number states of the five tumors sequenced. Four of the five tumors contain the CFA27 deletion. Middle: Copy-number states of additional six tumor genomes with the deletion detected by digital PCR. Right: ddPCR results for nine tumor genomes without deletion on CFA27. Errorbars indicate the 95% confidence limits as determined from the Poisson distribution.

A digital PCR assay was designed to confirm the data obtained by the DOC analysis and to screen 15 additional tumor genomes for the presence of the deletion. The deletion of the CFA27 locus was confirmed for the four tumors for which the deletion was initially detected by DOC analysis. Furthermore, the deletion was detected in six out of 15 additionally analyzed tumor genomes giving a total of 50% tumors carrying the deletion (Figure 4). No significant correlation between the deletion and tumor classification, stage, ER status, or *HER2* gene amplification was seen (Table 7). Nevertheless, Ki-67 scores were significantly higher when the CFA27 deletion was present (p<0.001; Wilcoxon U).

**Table 7:** Tumor CFA27 deletion status in conjunction with tumor class, stage, Ki-67-expression, ER-expression and *HER2* gene amplification status.

ID	Tumor Class	Stage	CFA27DEL	ki67	ER	HER2
T49	Complex carcinoma	T2NXMX	+	35	+	−
T47	Simple tubulopapillary	T1NXMX	+	30	+	−
T30	Osteosarcoma	T3NXM0	+	20	+	+
T52	Simple tubulopapillary	T1NXMX	+	30	+	−
T40	Simple tubulopapillary	T1N0M0	+	35	−	−
T69	Simple solid	T3NXM0	+	45	+	−
T73	Simple solid	T3N0MX	+	45	−	−
T81	Simple solid	T1N0M0	+	35	−	−
T86	Simple solid	T1N0M0	+	45	+	−
T108	Simple carcinoma	T3N0MX	+	ND	ND	−
T35	Simple tubulopapillary	T3NXM0	−	35	+	−
T60	Complex carcinoma	T1N0M0	−	ND	ND	+
T65	Complex carcinoma	T1N0M0	−	15	+	−
T78	Simple tubulopapillary	T1N0M0	−	10	+	−
T92	Carcinosarcoma	T3N0M0	−	10	+	−
T83	Simple tubulopapillary	T3N0M1	−	25	+	+
T98	Simple tubulopapillary	T3N0M0	−	ND	ND	+
T97	Simple tubulopapillary	T3N0M0	−	30	+	−
T99	Complex carcinoma	T3N0M0	−	10	+	−
T104	Simple tubulopapillary	T3N0MX	−	20	+	−

IHC results could not be determined for some tissues (labeled ND).

### Detection of Tumor Signatures in Cell-free DNA

**Figure 5:**
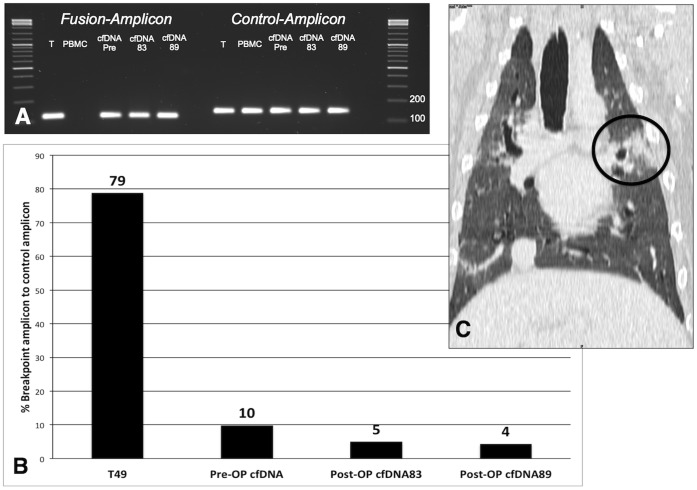
Tumor-derived cfDNA detection in the plasma of animal 49. Panel A: Agarose gel image of the breakpoint specific and control amplicon PCR for the various specimens obtained from animal 49. The breakpoint specific amplicon was not detected in the PBMC DNA. Panel B: Digital PCR results; mutated DNA content is displayed as breakpoint amplicon in percent of control amplicon. Panel C: Tomographic image of the lung of animal 49, the circle indicates the metastatic lesion.

For four sequenced tumor genomes we have designed unique PCR assays to detect specific rearrangements that were found in PEM analyses to span the chromosomal breakpoint. We could detect tumor-specific DNA in all four plasma samples taken prior to the surgical removal of the tumors. Also, the fraction of the rearranged fragment relative to the control was determined by digital PCR and varied substantially between the animals in both, the tumor and cfDNA (Table 8). Additional post-surgery blood samples were obtained from animal 49 and the cfDNA was analyzed. The fraction of the tumor-specific breakpoint was 11% just before surgery. 83 weeks after surgery the fraction was decreased to 5%, but had not reached zero, confirmed by a sample at 89 weeks that still contained 4.2% rearranged cfDNA. A subsequent tomographic examination of the animal revealed metastatic lesions in the lung explaining the persistent presence of the tumor-biomarker in the plasma (Figure 5).

**Table 8:** Content of tumor-specific rearrangements detected by droplet digital PCR.

	PBMC	Tumor	cfDNA(pre-surgery)
Animal 49	0	79%	10%
Animal 47	0	59%	1%
Animal 52	0	21%	11%
Animal 30	0	84%	24%

Percentages relative to the *FGF5* control fragment are shown.

### Representation of Copy-number Imbalances in the Plasma Cell-free DNA

**Figure 6:**
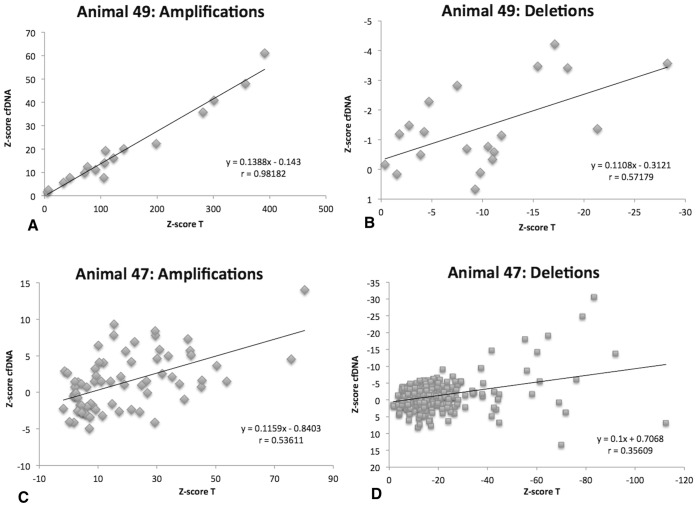
Z-Score distribution derived from paired-end sequencing of tumor and cfDNA specimen of two animals. Panel A: Z-Scores obtained for amplified regions in tumor 49 compared to the Z-scores obtained in the pre-surgery cfDNA. Panel B: Z-Scores obtained for deleted regions in tumor 49 compared to the Z-scores obtained in the pre-surgery cfDNA. Panel C: Z-Scores of amplified regions in tumor 47 compared to the Z-scores obtained in the pre-surgery cfDNA. Panel D: Z-Scores of deleted regions in tumor 47 compared to the Z-scores obtained in the pre-surgery cfDNA.

**Figure 7:**
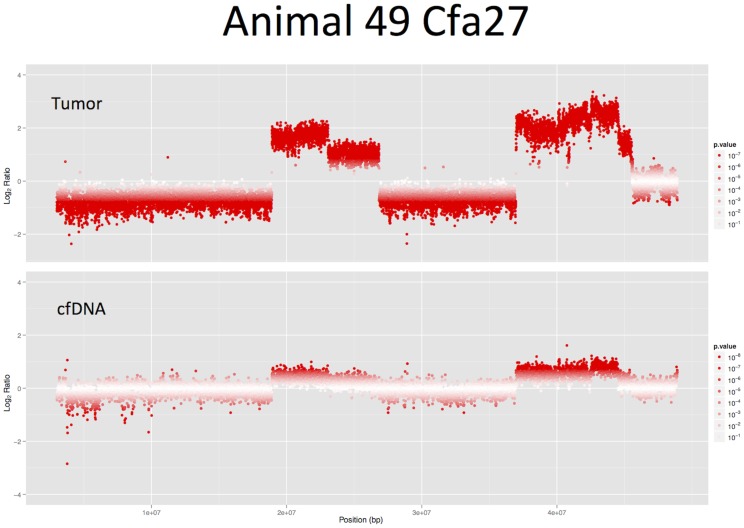
CFA27 CNV-seq results from paired-end sequencing of the tumor T49 and the pre-surgery cfDNA sample. The high amplifications detected in the tumor are also visible in the animal’s plasma cfDNA.

In addition to the mate-pair sequencing paired end libraries were prepared from two of the tumor genomes (T49 and T47) and corresponding cfDNA. The mapped reads were counted in sliding windows of 5 Mbp across all chromosomes and Z-scores. In order to estimate the reflection of tumor aneuploidies in the cfDNA we compared the Z-values of the two specimens in correlation scatterplots (Figure 6). The highest correlation between tumor and cfDNA was found for the amplified regions of tumor 49 (r = 0.98, p<0.01). Although less strongly correlated, the negative Z-scores for deleted regions in the tumor were also reflected in the cfDNA (r = 0.57, p<0.01). In animal 47 the Z-scores for amplifications were correlated with r = 0.53 (p<0.01) and deletions with r = 0.35 (p<0.01). These results are in concordance with the digital PCR assay for the tumor specific rearrangements where a ten-times higher content of tumor-derived DNA was detected in the plasma of animal 49 (11%) compared to animal 47 (1%). Therefore, the detectability of tumor-specific CNIs was higher in animal 49 and a stronger correlation of the tumor and cfDNA Z-scores was seen. Figure 7 illustrates the copy-number changes of the tumor and the pre-surgery cfDNA sample as detected by CNV-seq.

## Discussion

Mate-pair sequencing of five canine mammary carcinomas revealed heterogeneous patterns of genomic aberrations. Even the three tubulopapillary carcinomas did not show obvious commonalities in CNI patterns.

Genomic instabilities and mutations are considered an enabling characteristic for cancer development [57]. Genomic instabilities do manifest in different architectural types that were first described for colorectal cancers in humans, but are also found in other malignancies [58], [59]. The most abundant type of genomic instability is the chromosomal instability (CIN) phenotype that can be further divided into the whole chromosome aneuploidy and segmental aneuploidy subtypes [58]. Whole chromosome aneuploidy results from erroneous chromosome segregation during mitosis, while segmental aneuploidies also termed structural rearrangements/aberrations emerge from DNA strand breaks [58].

Two of the analyzed tumor genomes (T49 and T30) had high levels of structural aberrations at the subchromosomal level, while in one tumor (T47) chromosomal instability that affects whole chromosomes was seen. Hypoploidy as seen in this tumor is frequently observed in canine mammary gland tumors, but is uncommon in human breast carcinomas where amplifications especially of chromosomes HSA1q, HSA11q, HSA8q and HSA16p occur [60]–[63].

In the genome of tumor T49 highly complex interchromosomal rearrangements confined to only a few chromosomes are indicative of a chromothripsis event, a recently discovered phenomenon that describes a single catastrophic chromosome shattering event detected in 2–3% of human cancers [64]–[66]. The affected chromosomes CFA27 and CFA35 are syntenic to human chromosomes HSA12 and HSA6, respectively. While HSA6 is described to be frequently involved in complex amplification patterns in human breast cancer, HSA12 aberrations are less common [62]. The only detected fusion between two genes, *SOX5* and *ANO2* located on CFA27, occurred in T49. However, this fusion has not been described in human cancers and has most likely no functional relevance. Rather, the fusion - as many other gene aberrations in a cancer genome - is a passenger event that occurred due to gross genomic instability and represents a private structural variation.

The highest number of rearrangements and aneuploidies were detected in the genome of tumor T30. The pattern of CIN is indicative of a “mutator” phenotype. The mutator phenotype model proposes that chromosomal instability in cancer cells arises from a cascade of mutations in genes that maintain the genetic stability of a cell [64], [67]. Another hypothesis implies that chromosome aneuploidy is the cause of structural rearrangements. According to this hypothesis an unbalanced karyotype leads to an unbalanced expression of thousand of genes and also impairs the DNA break repair mechanisms leading to structural rearrangements and mutations [68]. Whether mutations in genes such as p53 or the early development of aneuploidies are the primary cause of cancer is still debated, but most probably none of the two closely associated phenomena is the exclusive primary cause of all cancers [69].

In the T52 tumor tissue only a few chromosomal breakpoints were detected by PEM signature analysis and DOC analysis revealed only low amplitude deletions and amplifications. Furthermore, the level of the breakpoint specific amplicon was only 21% in relation to the tested reference gene. This together with the relatively low coverage in the samples of this animal makes a CIN classification of this tumor not suitable. Similarly no clear copy-number imbalances were detected in the genome of T35. As previously described for human breast cancers a low percentage of tumors displays such “flat” profiles [62]. However, such a profile might also result from a high percentage of non-neoplastic cells in the specimen.

The patterns of CIN detected in canine mammary tumors were similar to the types of rearrangements described by Hicks and colleagues in human breast cancers [62]. With its primarily whole chromosome alterations tumor T47 was reminiscent of the simplex type, while T30 resembled the complex type I or “sawtooth” pattern and T49 belonged to the complex Type II or “firestorm” type [62]. These genomic architectural classes were extended into eight subgroups and it was shown that the level of complexity of aberrations is an independent prognostic marker [70]. Further research is needed to show whether there is a significant sub-group of canine tumors that display no gross amplifications or deletions.

Several well-described cancer genes were affected by chromosomal and segmental copy-number changes in the five different tumor genomes. Tumor T47 carried an additional copy of chromosome CFA1. Gains of CFA1 were also detected in studies of other dog cancers [71], [72] and the proto-oncogene *MYB* has been mapped to this chromosome [73]. *MYB* amplification has been found at high frequencies in human hereditary breast cancer. The protein is highly expressed in estrogen receptor positive breast tumors and the enhanced expression hinders apoptosis and differentiation of the cancer cells [74], [75].

Interestingly, the osteosarcoma T30 was the only tumor showing an amplification of CFA13 containing the oncogenes *cMYC* and *KIT.* cMYC amplifications and CFA13 gains have been detected in canine and human cancers before [76]–[78]. Interestingly, the tumor-suppressor gene *BRCA1* was slightly amplified in T30.

*FGFR1* is amplified in the osteosarcoma T30 but deleted in the tubulopapillary carcinoma T52. *FGFR1* amplification contributes to high metastatic potential and resistance to endocrine therapy of human breast carcinomas and is thought to be a major contributor to the poor prognosis of the luminal B subtype [48]. Nevertheless, a downregulation of FGFR1 expression has been previously described for canine metastatic mammary carcinomas as compared with non-metastatic and normal mammary tissue [79].

*PTEN* located on chromosome CFA26, was also deleted in the T30 genome, which is in line with earlier reports of human and canine mammary gland cancers [80]. This tumor was the only one sequenced harboring a *HER2/ERBB2* amplification. As assessed by ddPCR, four out of twenty tumors carried a *HER2/ERBB2* amplification, which is consistent with data for human breast cancer, where the amplification has an incidence of 15–25% [13]. HER2/ERBB2 overexpression is detected in canine mammary tumors to varying extends (18–74%). Results seem to depend on the method of detection [11], [81], [82], since methods (IHC, FISH, qPCR) do not always correlate perfectly [83], [84]. In addition T30 carried a deletion of chromosome 22, on which the retinoblastoma-associated protein (*RB1)* gene is located. Together, with the deletion of the *RB1* regulator genes *CDKN2A-CDKN2B* in two of the other samples the Rb pathway was affected in 60% of the analyzed tumors. The high prevalence of abnormalities of genes of the Rb pathway has been described for canine malignancies elsewhere [76], [85].

Taken together the genomic aberrations seen in the five tumor genomes are heterogeneous and show similarities as well as differences to the conditions described in human breast cancers. Based on former comparisons between canine and human CNI a total overlap of the affected genomic regions cannot be expected. Some recurrent CNIs are species-specific because they arise from evolutionary unstable hypervariable regions as found for example on human chromosome 8p23.1 [23].

The chromosome CFA27 was found entirely deleted in two of the sequenced tumor genomes, while in two others segmental deletions were detected. The recurrent proximal deletion of CFA27 was confirmed by ddPCR and found in 10 out of 20 tumor genomes. Screening for cancer-associated genes in this region revealed *PFDN5* as most probably affected by the recurrent deletion. *PFDN5* - also termed cMYC modulator MM1– is described as a tumor suppressor that acts by repressing the expression of the cMYC oncogene product [86]. A lowered expression of the gene was found in progestin-induced canine mammary hyperplasia [87]. This is the first report showing that the gene is recurrently deleted in canine tumors. Future studies are needed to determined whether the recurrent *PFDN5* deletion can be confirmed in a larger group and whether this deletion is specific to mammary tumors. The deletion was not associated with ER positivity or HER2 amplification, but showed correlation to the KI-67 index. The observed a high prevalence of ER-positive malignant tumors in our small study group is in contrast to other reports [14], [88], [89]. However, a wide variety of ER-positivity in canine mammary cancers ranging from 10% to 87.5% has been reported and reasons for this are discussed elsewhere [89]. The high percentage found in our study group may be due to the high binding affinity towards the canine ER of the used antibody, which therefore leads to a higher detection rate.

In addition to the analysis of the tumor genomes, the cfDNA of the animals was examined. Tumor-specific breakpoint PCR assays were developed for four of the five tumors. For the first time in dogs the occurrence of tumor specific chromosomal breakpoints was quantitatively measured in the plasma. The amplification of fusion points from the cfDNA is known to be a powerful method for tumor recurrence monitoring [34], [35]. Remarkably, we were able to detect the persistence of the plasma tumor marker after surgery in one dog. A consequently scheduled tomographic examination revealed metastatic lesions in the lung. Leary and colleagues have shown similar results in humans [34]. Unfortunately, post surgery blood samples could not be obtained for the other animals enrolled in the study. However, the current study reinforces the suitability of cfDNA as tumor marker. The elaborate and costly detection of individual chromosomal breakpoints and the need for initial sequencing of the tumor genome pose significant drawbacks of such an approach. Furthermore, tumor recurrence monitoring is not commonly conducted in canine mammary cancer patients. But the technology envisaged herein for canine patients provides the basis for further studies on the feasibility of cfDNA as biomarker for drug response monitoring and diagnostics. The amplification of chromosome breakpoints is a highly specific assay, because in theory the breakpoint is not amplified from non-neoplastic body cells; linking the presence of the amplicon directly to the presence of cancer cells. But, as also shown herein quantitative copy-number differences in the cfDNA are explained by the same copy-number alterations occurring in the tumor. The cfDNA and tumor genomes of two animals (49 and 47) were paired-end sequenced and the copy-number aberrations in both specimens were evaluated by Z-score analysis. A good concordance of tumor and cfDNA CNIs was achieved for the regions with high-level amplifications. As shown by Chan and colleagues for human cancer patients the detectability of tumor-associated CNIs in the cfDNA depends on the fraction of tumor DNA in the plasma and the depth of sequencing coverage [36].

With the advance of more cost-effective digital PCR instrumentation with a high inherent sensitivity and precision it appears possible to detect such subtle quantitative differences in cfDNA samples directly. Similar approaches have been described for the detection of fetal aneuploidies from the maternal blood [90]. Furthermore, knowledge about recurrent copy-number changes in canine and human cancers is constantly growing [23], [91], [92]. Adding to this knowledge is our finding of a newly discovered recurrent deletion on CFA27 in canine mammary tumors. On this basis it seems reasonable to assume that cost-effective copy-number based assays for tumor diagnostics from a blood sample can be developed in the near future.

## Supporting Information

Table S1 **Description, position and primer sequences for the chromosomal breakpoints used to detect and quantify tumor specific cfDNA in the plasma.**
(XLSX)

Table S2 **Chromosomal regions with detected copy number aberrations and a listing of refSeq annotated in these regions for T30.**
(XLSX)

Table S3 **Chromosomal regions with detected copy number aberrations and a listing of refSeq annotated in these regions for T47.**
(XLSX)

Table S4 **Chromosomal regions with detected copy number aberrations and a listing of refSeq annotated in these regions for T49.**
(XLSX)

Table S5 **Chromosomal regions with detected copy number aberrations and a listing of refSeq annotated in these regions for T52.**
(XLSX)

Table S6 **Listing of all validated chromosomal breakpoints together with the primer sequences used for validation.**
(XLSX)

Skript S1 **The file contains the perl script used for filtering the structural variation data.**
(TXT)
